# Dietary habits and cognitive performance in primary school students: a cross-sectional study in Khemisset region in Morocco

**DOI:** 10.3389/fnut.2025.1643854

**Published:** 2025-10-10

**Authors:** Mohcin Elkhatir, Chaymae Ghaffouli, Bouchra Louasté, Abdechahid Loukili, Youssef Aboussaleh

**Affiliations:** ^1^Biology and Health Laboratory, Faculty of Sciences, Ibn Tofail University, Kenitra, Morocco; ^2^Biotechnology, Environment, Agri-food and Health Laboratory, Faculty of Sciences Dhar El Mahraz, Sidi Mohammed Ben Abdellah University, Fez, Morocco

**Keywords:** cognitive performance, dietary habits, survey, school-aged children, Morocco

## Abstract

**Background:**

In children, cognitive performance is influenced by dietary habits, health status, and socioeconomic factors. Poor diets, especially high intakes of ultra-processed foods, together with psychological stress and learning difficulties, can impair attention and concentration in school-aged children. It is critical to comprehend these interactions. It supports improved long-term health and educational outcomes.

**Methods:**

A cross-sectional survey was conducted among primary school students in the region of Khemisset, Morocco. Socioeconomic parameters, dietary habits, health status, visual problems, psychological stress, and learning difficulties were assessed via questionnaires completed with parental consent. Cognitive performance was assessed via the d2R test, which measures processing speed, concentration, and accuracy.

**Results:**

Of the 330 students (53.9% girls, 46.1% boys), the majority had a BMI of less than 18.5 (59.7%). Most reported no health problems (91.2%) or vision problems (89.4%), while 32.7% experienced psychological stress and 43% had learning difficulties. In terms of dietary habits, 66.9% of participants ate three or more meals per day, but 60% skipped breakfast. The d2R test results revealed that higher meal regularity and breakfast consumption were associated with better processing speed, concentration, and accuracy.

**Conclusion:**

This study highlights the significant influence of dietary habits and lifestyle factors on cognitive performance among primary school students. Regular meal consumption, particularly breakfast, was positively associated with increased attention, concentration, and processing accuracy. In contrast, high intake of ultra-processed foods and the presence of psychological stress and learning difficulties were linked to poorer cognitive outcomes.

## 1 Introduction

In recent years, global food systems have experienced a substantial shift in consumer preferences toward healthier and more sustainable food choices. Consumed food can be a source of nourishment, as it can cause disease and mental health disorders (1). In recent years, researchers have demonstrated that proper nutrition can help reduce the symptoms of various mental disorders (2). Moreover, the use of biotechnology and genetic modifications in agriculture (3) and the drastic use of pesticides (4) may be contributing factors to the development of mental illness.

Childhood represents a profoundly critical period, not only for robust physical growth but also for the intricate and foundational development of the brain (5, 6). This period lays the groundwork for cognitive abilities, emotional regulation, and social skills that will influence an individual’s entire life (7, 8). During this stage, the brain requires a steady supply of essential nutrients to support cognitive functions such as attention, memory, learning, and problem-solving (9, 10). Adequate dietary habits established during early years can have lasting impacts on a child’s health, academic performance, and overall wellbeing (11, 12).

An appropriate diet provides not only physical benefits but also mental benefits, which in turn promote overall wellbeing. These benefits often manifest themselves indirectly and are sometimes unknown but remain essential (13, 14). The choice of food involves the consumption of untreated fruits, vegetables, cereals, and legumes. Foods that are rich in nutrients such as vitamins, minerals, and antioxidants have beneficial effects on the brain, helping to improve mood, concentration, and other cognitive functions (15, 16). An unhealthy diet has been linked to various mental health problems, including anxiety, depression, and attention disorders (17, 18). It can thus be concluded that an improvement in mental health can be expected by avoiding processed food products (19).

Human cognitive function is influenced by a variety of environmental factors, among which diet and nutrition play a central role, a fact that is increasingly recognized (20, 21). The brain, as a metabolically active organ, requires a constant and adequate supply of nutrients to maintain optimal performance. A range of studies have demonstrated that dietary patterns rich in essential nutrients such as omega-3 fatty acids, antioxidants, vitamins, and minerals can facilitate cognitive processes, including memory, attention, and processing speed (22–24). Conversely, diets characterized by elevated levels of saturated fats, refined sugars, and ultraprocessed foods have been consistently associated with cognitive decline, attention deficits, and an increased risk of neurodegenerative conditions (25, 26).

Although this large-scale situation is the result of a complex interaction between lifestyle habits and socioeconomic factors, it is currently well accepted that dietary imbalance, particularly caloric intake that exceeds energy expenditure, represents a major contributor to the development of metabolic and cardiovascular disorders (27, 28). Indeed, the increasing consumption of ultra-processed products, rich in fats and “added” sugars, has been shown to negatively affect the body’s metabolic homeostasis over time, with downstream effects on cognitive function (29, 30).

While the relationship between diet and cognitive function has been the subject of extensive research in high-income countries, there has been a comparatively limited amount of research conducted in North African contexts, particularly among schoolchildren in rural Moroccan communities. It is imperative to understand the manner in which local dietary patterns influence children’s cognitive abilities to formulate efficacious school health policies and interventions that are adapted to the region’s cultural and socioeconomic realities.

Social cognitive theory, which highlights how behavioral, personal, and environmental factors influence behavior, serves as the foundation for this study. This framework is especially useful for comprehending how dietary decisions affect children’s cognitive development. The target population involved primary school students who were willing to provide information regarding their food consumption and academic performance. The research question for this study is as follows: What is the relationship between students’ dietary habits and attention patterns as measured via the d2R test? In this context, the objective of this study was to identify the impact of certain socioeconomic parameters, as well as certain dietary habits such as sugar consumption, on attention and concentration among primary school students in the Ait Yadine region in Khemisset, Morocco. Furthermore, given the importance of diet in cognitive development, this study aims to explore the relationships between dietary habits and various domains of cognitive performance among primary school students.

## 2 Materials and methods

### 2.1 Data collection and study area

**Figure 1:**
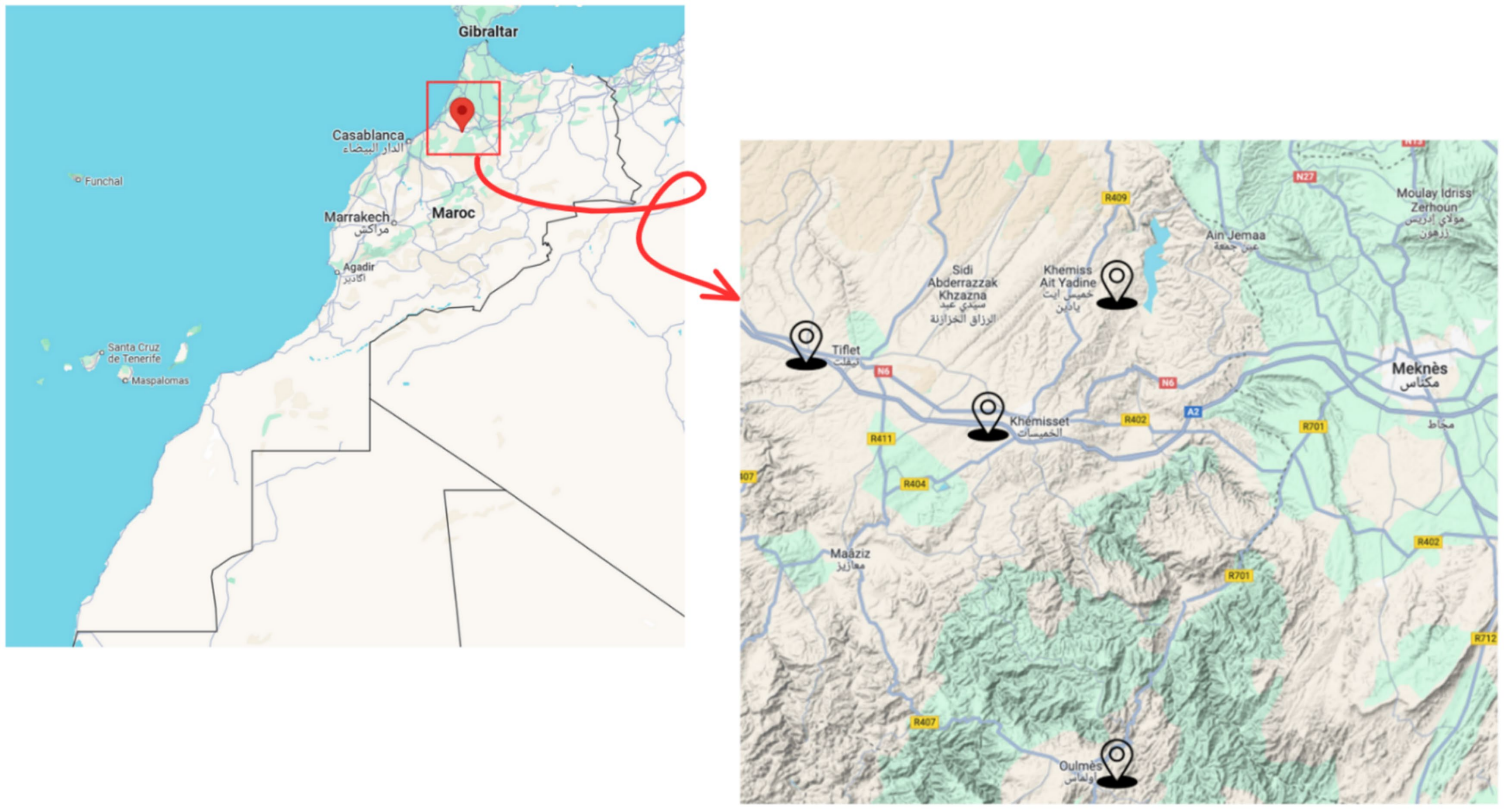
The study area.

This research constitutes a prospective cross-sectional survey that was carried out among schoolchildren in the Aït Yadine region, which is located in the province of Khemisset in Morocco, between early January and May 2025 (Figure 1). The study population comprises a total of 325 schoolchildren from both urban and rural areas, reflecting diverse socioeconomic conditions and lifestyle patterns. Schools were sampled using a stratified random approach, and children were selected from class lists according to predefined inclusion/exclusion criteria; all participants were enrolled after parental informed consent. Khemisset is distinguished by its heterogeneity in terms of income levels, educational attainment, and access to fundamental services and offers a pertinent context for the examination of population-level health and developmental indicators. While the study sample was limited to primary school students, the demographic characteristics of the participants (age, gender distribution, and socioeconomic status) are broadly consistent with those reported in national statistics for rural Moroccan schoolchildren. However, given the regional focus, the findings may not be fully generalizable to all Moroccan children, particularly those in urban areas.

### 2.2 Study design

The survey was designed as a structured questionnaire divided into three main sections to comprehensively evaluate the associations among nutritional patterns, individual health status, and cognitive performance. The initial section of the questionnaire comprised questions pertaining to sociodemographic and health-related information, including sex, age, weight, and height, to calculate body mass index (BMI). Additionally, students were asked to indicate the presence of any chronic health problems, vision difficulties, psychological stress, or learning difficulties. The psychological stress of the participants was measured via a self-reported item that was developed for the specific purpose of this study. The students were invited to respond to an enquiry regarding the presence or absence of feelings of stress, worry, or emotional discomfort in the preceding weeks. To facilitate comprehension, the concept of “psychological stress” was explicated in a manner commensurate with the age of the subjects by trained data collectors. Illustrative examples included feelings of anxiety prior to a test or feelings of being overwhelmed by scholastic tasks. Information on learning difficulties was collected through self-reports from the participants or their guardians. The participants were asked whether they experienced challenges in learning, reading, writing, or concentrating in class.

Furthermore, the study aimed to ascertain the level of engagement of the participants in regular physical activity. The second section focused on dietary habits, assessing the number of meals consumed per day (including breakfast), breakfast consumption, the frequency and timing of snacking (between breakfast and lunch or between lunch and dinner), and the primary food groups commonly consumed (e.g., fruits and vegetables, dairy products, protein-rich foods, grains, fats, and processed foods). The classification of food categories was based on the Moroccan National Nutrition Strategy guidelines (31) (or FAO/WHO food-based dietary guidelines), which methodically categorizes foods into major groups. The grouping of food items was based on their nutritional characteristics and consumption patterns, using the NOVA classification framework (32). The NOVA classification system categorizes foods according to the extent and purpose of processing. Alternatively, in the event that the grouping was in accordance with a national or WHO dietary guideline, this should be specified.

The third section of the study comprised the standardized d2R test, which was utilized to evaluate students’ attention and concentration capacities, thereby enabling an objective assessment of their cognitive performance. The questionnaire was completed with parental consent, and all data were collected in a classroom setting under standardized conditions.

### 2.3 Anthropometric measurements

Weight and height were measured according to the standard norms. During the measurement, the participants were in their underwear, without shoes. The weight was determined via a new mechanical scale (Terraillon, with an accuracy of 0.5 kg). Height was measured using a height chart with an accuracy of 0.1 cm. Body mass index (BMI), kg/m^2^, was calculated by dividing weight in kg by height squared in m^2^. To account for age and sex differences in children, BMI-for-age Z-scores were calculated based on the WHO Child Growth Standards (33). These Z-scores allow for the classification of nutritional status (underweight, normal weight, overweight, and obese) in a way that is appropriate for children’s growth patterns.

### 2.4 Cognitive measure (d2R test)

The d2R attention test measures visual scanning ability, processing speed, and degree of accuracy, regardless of intelligence level. According to Bates and Lemay (34), the d2R test has received extensive validation for use with children and teenagers. The test consists of 47 interspersed target and distractor characters × 14 lines, and the respondents’ task is to cancel as many target characters as possible while ignoring the distractor characters. Standard test results were included in the trial, namely: “processing speed” (TN; total number of characters processed) and two overall performance measures: “total performance” (TN-E; total number of processed characters minus total committed errors) and “concentration performance” (CP; total number of correctly canceled target characters minus commission errors). Additionally, there are two types of errors: “errors of omission” (E1; unmarked target characters), “errors of commission” (E2; incorrectly canceled distractor characters), and “total errors” (E total; the sum of E1 and E2). The tests were conducted in classrooms between 9 a.m. and 11 a.m. Environmental factors such as noise were rigorously controlled by the study team and teachers.

The d2R test (performance test) allows us to obtain three values:

- CCT: processing rate (BZO), i.e., the number of target objects processed line by line.
- CC: concentration capacity (KL), i.e., the number of objects correctly crossed out, which is determined by subtracting commission and omission errors from total target hits (KL = BZO − AF − VF).
- E%: accuracy (F%), i.e., F% = ((AF + VF)/BZO) × 100.

### 2.5 Data analysis

The data are presented as the means and standard deviations (SDs) or as numbers (*n*) and percentages (%). Differences between the intervention groups were assessed by a one-way ANOVA for continuous variables and the chi-squared test for categorical variables.

Analyses of the associations between the results of the d2 test (dependent) and the dietary food consumed (independent) were carried out with linear mixed effects models with a school class as a random intercept. Unadjusted models and models adjusted for age, sex, parental education level, and physical activity were constructed for the d2R test results.

Two-sided *p*-values of less than 0.05 were considered statistically significant. Analyses were performed using the Statistical Package for the Social Sciences (SPSS, version 22).

## 3 Results and discussion

### 3.1 Participant characteristics

The study sample consisted of 330 primary school students, with a slightly greater proportion of girls than boys (Table 1). The majority of participants were aged between 11 and 12 years, followed by those aged between 13 and 14 years, whereas students aged between 9 and 10 years and those aged between 15 and 17 years represented smaller fractions. Regarding the nutritional status of the students, 59.7% of the participants were classified as undernourished on the basis of BMI-for-age Z-scores, with Z-scores below −2 standard deviations (SDs) from the median. Approximately 40% of the children fell within the normal nutritional range (Z-scores between −2 and +1 SD), whereas only 0.3% were identified as overweight (Z-scores > + 1 SD). No cases of obesity (Z-scores > + 2 SD) were observed. Nevertheless, undernutrition remains a significant problem in low- and middle-income countries, where socioeconomic disparities frequently result in insufficient dietary intake (35, 36).

**Table 1:** Characteristics of the sample population.

Characteristics	*n*	%
Gender		
Boy	152	46.1
Girl	178	53.9
Age		
9–10	41	12.4
11–12	146	44.2
13–14	91	27.6
15–16	42	12.7
17	10	3
Body mass index		
<18.5	197	59.7
18.5–24.9	132	40
25–29.9	1	0.3
>30	0	0
Health problems		
Yes	29	8.8
No	301	91.2
Trouble vision		
Yes	35	10.6
No	295	89.4
Sport		
Yes	251	76.1
No	79	23.9
Psychological stress		
Yes	108	32.7
No	222	67.3
Learning difficulties		
Yes	142	43
No	188	57

The health status assessment revealed that 91.2% of the students reported no health problems, whereas 8.8% reported having a health condition. Visual impairments were reported in 10.6% of the sample. With respect to physical activity, 76.1% of students indicated regular engagement in sports or exercise. Furthermore, 32.7% of students reported experiencing psychological stress, and 43% reported having learning difficulties. The results suggest that participants generally perceived their physical health to be good, with relatively low rates of reported illnesses and visual impairments. The high level of physical activity is encouraging; however, the considerable proportions of students reporting psychological stress and learning difficulties raise concerns about mental wellbeing and academic support needs within this population.

Psychological stress during childhood is recognized as a major risk factor for both mental health disorders and impaired cognitive development (37, 38). Similarly, learning difficulties may be attributed to several interacting factors, including nutritional deficiencies, socioeconomic challenges, and educational quality (39, 40).

The findings provide critical baseline information on the sociodemographic and health characteristics of the student population, which is essential for understanding the factors influencing cognitive performance and wellbeing.

**Figure 2:**
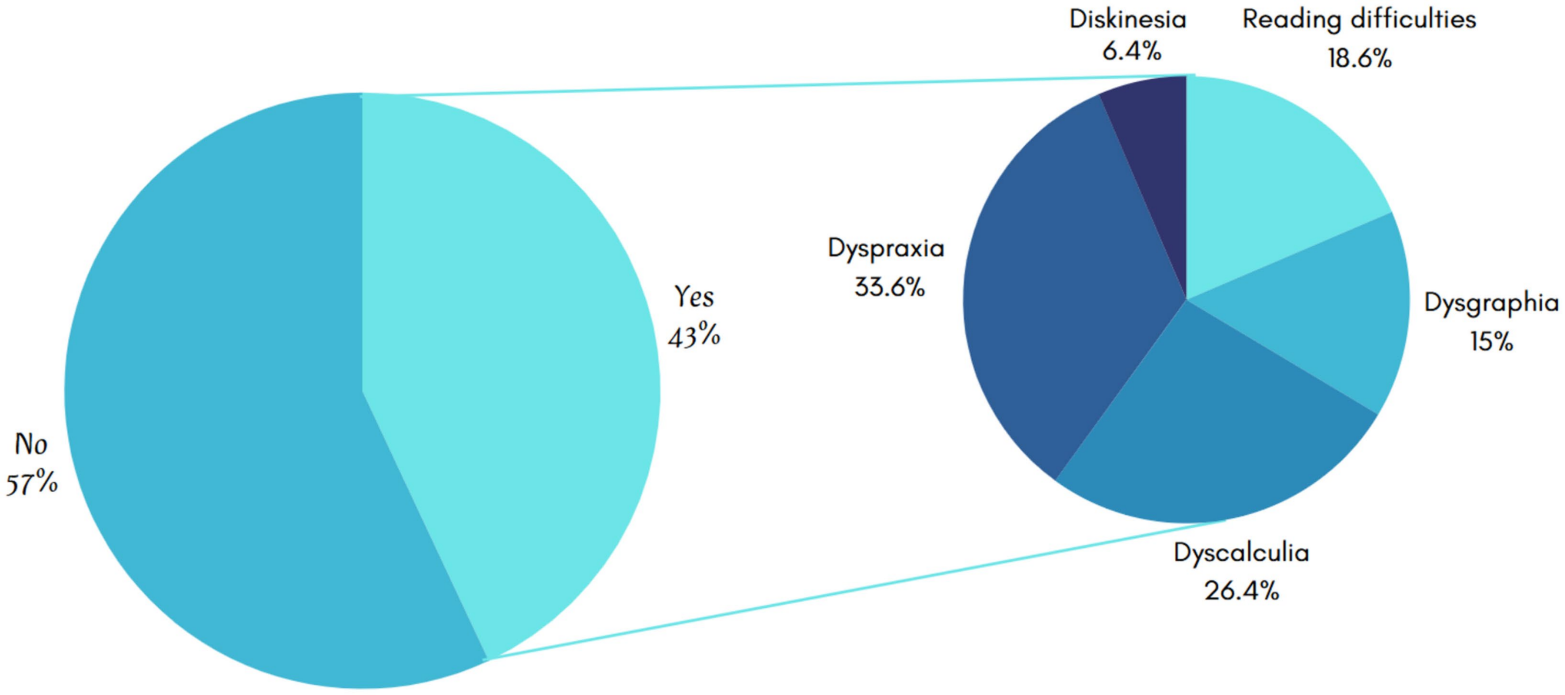
Prevalence and types of learning difficulties among primary school students.

Among students with learning difficulties (Figure 2), dyspraxia was the most commonly reported issue. Dyspraxia, a developmental coordination disorder, has been shown to interfere with motor skills and academic performance, particularly in tasks requiring fine motor control, such as writing (41, 42). Dyscalculia was the second most prevalent condition. Dyscalculia, a condition characterized by difficulties with numerical concepts and arithmetic, is increasingly recognized for its impact on students’ academic progression and self-esteem (43).

Reading difficulties, which are frequently associated with dyslexia, constituted 18.6% of the cases. This finding is consistent with the extensive documentation of the role of phonological processing deficits in academic underachievement (44). Dysgraphia constitutes a significant impediment, particularly with respect to written expression and composition skills. Finally, dyskinesia was reported in 6.4% of students. Although less common, dyskinesia, involving involuntary muscle movements, has been demonstrated to hinder school participation and fine motor tasks to a considerable degree (45).

### 3.2 Dietary patterns

A comprehensive understanding of the relationships of meal frequency, breakfast consumption, and snacking habits with body mass index (BMI), health problems, vision problems, learning difficulties, and psychological stress provides valuable insights into dietary patterns and their potential impact on health (Table 2).

**Table 2:** Distribution of meal frequency, breakfast consumption, and snacking habits according to gender, BMI, health status, vision problems, learning difficulties, and psychological stress.

Food consumption	Gender		Body mass index			Health problems		Trouble vision		Learning difficulties		Psychological stress	
	Girl	Boy	<18.5	18.5–24.9	25–29.9	Yes	No	Yes	No	Yes	No	Yes	No
Number of meals per day													
2	6	3	5	4	0	0	9	0	9	4	5	3	6
3	113	108	126	94	1	17	204	25	196	99	122	66	155
4	40	27	41	26	0	7	60	5	62	26	41	24	43
5	19	14	25	8	0	5	28	5	28	13	20	15	18
Having breakfast													
Yes	47	50	141	91	1	18	215	27	206	97	136	70	163
No	131	102	56	41	0	11	86	8	89	45	52	38	59
Snacking													
Between breakfast and lunch	39	125	166	97	1	25	239	31	233	110	154	94	170
Between lunch and dinner	139	27	31	35	0	4	62	4	62	32	34	14	52

The majority of participants consumed three meals per day, whereas a smaller proportion reported consuming four or five meals per day. The prevalence of underweight individuals (BMI < 18.5) was highest among those consuming three meals per day (126 children), whereas those in the normal BMI range (18.5–24.9) were distributed across different meal frequencies, with a notable presence in the three-meal group (94 children). Overweight individuals (BMI 25–29.9) were more common among those who consumed 3–5 meals per day, demonstrating that meal frequency alone is not a direct predictor of BMI status but may interact with other dietary and lifestyle factors.

Regarding health problems, those without chronic conditions were more likely to eat three meals per day (204 children) than those with health problems (only 17 children in this group). A similar trend was observed in individuals without vision troubles, learning difficulties, or psychological stress, who also exhibited a greater tendency to consume three or more meals daily. These results suggest a potential correlation between regular meal consumption and overall wellbeing. Regular meal consumption plays a crucial role in maintaining physical health, cognitive function, and psychological wellbeing. The findings from this study align with those of Cabiedes-Miragaya et al. (46), who reported that individuals who maintain consistent meal patterns, including breakfast and regular snacks, exhibit better overall health outcomes.

Breakfast consumption was reported by 47 girls and 50 boys, with a notable proportion of participants exhibiting normal BMI (141 children) and underweight status (91 children). The majority of individuals who skipped breakfast had lower BMI values and were more likely to experience psychological stress and learning difficulties. These findings are consistent with prior research suggesting that breakfast consumption plays a crucial role in cognitive performance, metabolic regulation, and emotional stability (47, 48).

The habitual consumption of snacks significantly varied among the study participants. The majority of the subjects consumed snacks between breakfast and lunch, whereas a smaller proportion consumed snacks between lunch and dinner. Individuals who engaged in snacking between breakfast and lunch presented a greater prevalence of a normal BMI (166 children) than did those who snack later in the day. Interestingly, snacking before lunch was more prevalent among individuals without learning difficulties and those without psychological stress. These results highlight the potential role of morning snacks in maintaining energy levels and cognitive function. Many studies have indicated that distributing energy and nutrient intake across 4–5 eating occasions per day (rather than across three standard meals) has the potential to positively impact human health (49).

### 3.3 Cognitive outcomes

The data in Table 3 indicate an imbalance in the consumption of food groups among the participants. Protein-rich products, dairy products, and fats and oils were the most consumed, while the intake of fruits, vegetables, and grains was markedly lower. A substantial proportion of students also reported regular consumption of sugary and processed foods. The observed dietary pattern indicates a nutritional imbalance, characterized by excessive consumption of energy-dense and high-fat foods and insufficient intake of plant-based, fiber-rich options. Such trends give rise to concerns regarding the long-term implications for metabolic health and cognitive development. These findings underscore the necessity for school-based interventions aimed at promoting healthier and more balanced dietary habits among primary schoolchildren.

**Table 3:** Distribution and relative significance of consumed food groups among students.

Food groups	*n*	%	Absolute percentage significance (%)
Fruits and vegetables	123	10	39.8
Dairy products	252	20.5	81.6
Protein-rich products (meats, fish, eggs, etc.)	277	22.5	89.6
Grains et starchy foods	107	8.7	34.6
Fats and oils	253	20.6	81.9
Sugary and processed foods	218	17.7	70.6
Total	1,230	100.0	398.1

**Figure 3:**
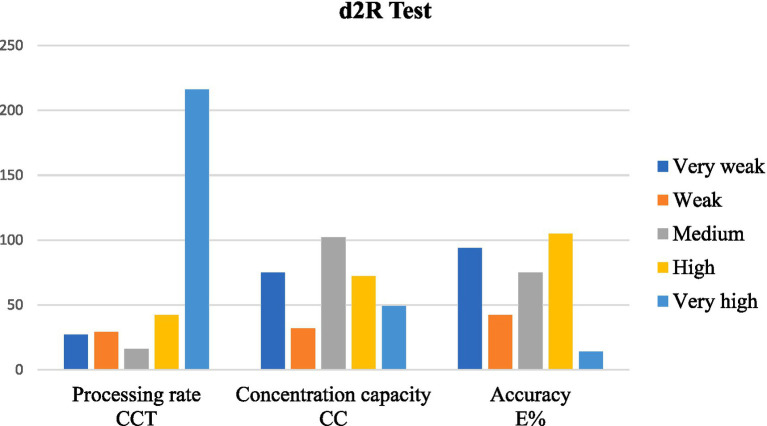
Cognitive performance profiles based on d2R test results.

The bar chart presents data on three variables, i.e., the CCT (processing rate), CC (concentration capacity), and E% (accuracy), categorized into five levels of performance: very weak, weak, medium, high, and very high (Figure 3). The CCT is predominantly represented by the “Very High” category, which has a significantly higher value than the other levels, whereas the “Medium” category has the lowest value. For CC, the highest values are observed in the “Medium” and “High” categories, followed by “Very High,” whereas the “Weak” and “Very Weak” categories remain lower. The “Very High” and “High” levels demonstrate the greatest values, while the “Very Weak” category is notably lower. The chart suggests that individuals with a higher processing rate (CCT) are overwhelmingly categorized as “Very High,” while concentration capacity (CC) and accuracy (E%) show a more balanced distribution across categories. This pattern may indicate that processing speed differs more significantly among individuals compared to concentration and accuracy.

Cognitive performance is affected by dietary patterns, with macronutrient composition playing a critical role in information processing, attention, and accuracy. The present study evaluated the impact of different food groups on cognitive function using three parameters: processing rate (CCT %), concentration capacity (CC %), and accuracy (E %) (Table 4). The findings revealed statistically significant differences among the food groups (*p* < 0.001), except for two food groups: fruits and vegetables and grains and starchy foods, underscoring the varied contributions of macronutrients to cognitive efficiency.

**Table 4:** Association between respondents’ dietary intakes and test d2R variables.

Food groups	CCT (processing rate) (%)					*p*-value	CC (concentration capacity) (%)					*p*-value	E% (accuracy) (%)					*p*-value
	Very weak	Weak	Medium	High	Very high		Very weak	Weak	Medium	High	Very high		Very weak	Weak	Medium	High	Very high	
Fruits and vegetables	1.62	6.5	6.5	13	72.35	>0.001	28.45	7.31	34.95	21.13	8.16	>0.001	28.45	15.45	22.76	30.08	3.25	>0.001
Dairy products	1.98	5.15	3.96	13.19	75.69	<0.001	42.28	5.55	30.15	25.40	18.25	<0.001	2.22	10.71	24.60	37.70	22.22	<0.001
Protein-rich products (meat, fish, eggs, etc.)	5.05	6.5	4.7	12.10	70.76	<0.001	20.6	7.58	30.32	24.19	17.33	<0.001	23.83	11.91	23.83	35.38	5.05	<0.001
Grains and starchy foods	1.87	7.48	9.35	14.95	66.35	>0.001	31.77	6.54	30.84	20.56	10.28	>0.001	30.84	16.82	24.30	23.36	4.67	>0.001
Fats and oils	1.98	5.14	4.74	14.62	73.52	<0.001	19.37	6.32	31.62	24.9	17.79	<0.001	22.92	9.09	25.3	38.73	3.95	<0.001
Sugary and processed foods	2.29	5.50	3.21	13.99	75	<0.001	18.35	6.42	28.44	27.52	19.27	<0.001	20.64	9.17	23.39	42.20	4.58	<0.001

For only the CCT (processing rate), across all the food groups, the majority of the respondents fell into the very high-level category, ranging from 66.35% (grains and starchy foods) to 75.96% (sugary and processed foods). The proportion of individuals in the “Very Weak” and “Weak” categories is relatively low, indicating that most people achieve a high processing rate.

In terms of processing speed, 72.35% of the respondents who consume fruits and vegetables fell into the very high level. This result is consistent with the findings of previous studies that emphasized the neuroprotective effects of polyphenols, flavonoids, and antioxidants, which are abundant in plant-based foods (50, 51). However, the distribution of concentration capacity is more balanced, with 34.95% in the medium category and 21.13% in the high category, suggesting a moderate effect on sustained attention. The accuracy performance remains relatively strong, with 30.08% achieving high accuracy, supporting the role of dietary antioxidants in cognitive maintenance.

Dairy products have the highest percentage of people in the very high processing rate category, which suggests that they may help with cognitive processing. However, 42.28% of the participants had very weak concentration capacity, suggesting that, while dairy products may facilitate short-term cognitive efficiency, their effect on sustained attention remains unclear. The accuracy scores show a more positive trend, with 37.70% in the high category, which supports previous findings that dairy-derived bioactive peptides and calcium play a role in neuronal function (52, 53). Compared with the other food groups, the very weak category accounts for 5.05% of the food group, which is slightly elevated.

Protein-rich foods, including meat, fish, and eggs, are strongly related to cognitive efficiency, with 70.76% of individuals falling into the very high category for processing rate. This phenomenon is likely mediated by the role of amino acids in the synthesis of neurotransmitters, especially dopamine and serotonin, which are essential for cognitive function (23). Furthermore, the distribution of concentration capacity is notable, with 30.32% of individuals falling into the medium category and 24.19% falling into the high category, suggesting a moderate enhancement in sustained attention. The accuracy scores substantiate these observations, with 35.38% attaining high accuracy, thereby suggesting that protein consumption fosters cognitive speed and precision. The variability may be indicative of disparities in protein sources, as research indicates that lean proteins and omega-3-rich fish are more advantageous for cognitive function than processed meats are (54, 55).

The impact of grains and starchy foods on cognitive performance appears to be less pronounced than that of other food groups, with 66.35% of children in the very high processing rate category, the lowest among all groups. The concentration capacity scores indicate a balanced effect, with 30.84% of individuals falling within the medium category and 20.56% falling within the high-level category. These findings suggest that grain-based diets may offer certain cognitive benefits, potentially influenced by fiber and micronutrient contents (56).

Fats are strongly correlated with processing speed, with 73.52% of individuals in the very high category. The role of dietary fats appears substantial. However, the observed effects may be contingent on the quality of the consumed fats. For example, the ingestion of unsaturated fatty acids (e.g., olive oil and omega-3 s) has been associated with cognitive benefits, whereas trans and saturated fats have been linked to reduced mental processing (57, 58). The distribution of the concentration capacity remains balanced, with 31.62% falling within the medium category and 24.9% falling within the high category. These findings serve to reinforce the role of essential fatty acids in cognitive endurance. The accuracy parameter also corroborates these benefits, with 38.73% achieving high accuracy, thereby supporting the findings of previous studies on the impact of healthy fats on executive function and memory (59, 60).

Sugary and processed foods represented an important proportion of individuals in the very high category for processing rate, suggesting an initial cognitive boost. However, an examination of accuracy scores reveals a disproportionate increase, with a mere 4.58% attaining very high accuracy. This finding suggests that, while sugar may temporarily increase cognitive speed, it does not contribute to long-term accuracy or attention. However, short-term energy increased from sugars can enhance cognitive performance, and excessive consumption is associated with cognitive decline and metabolic dysregulation (61, 62).

Overall, these findings underscore the importance of dietary choices in maintaining cognitive health and underscore the need for nutritional strategies that are aimed at optimizing brain function and reducing the risk of cognitive decline. Moreover, it has been demonstrated that adherence to the Mediterranean diet is associated with improved cognitive function and a reduced risk of cognitive decline. The emphasis of the diet on fruits, vegetables, whole grains, and healthy fats contributes to these cognitive benefits (63, 64).

## 4 Conclusion

The present study underscores the intricate interplay among dietary habits, socioeconomic factors, health status, and cognitive performance among schoolchildren. The present study revealed a high prevalence of nutritional imbalances, characterized by low consumption of fruit and vegetables and elevated intake of protein-rich, fatty, and processed foods. It is hypothesized that dietary patterns, which have been previously linked to long-term metabolic disturbances, may partially account for the observed cognitive disparities.

Furthermore, approximately 50% of the students reported experiencing learning difficulties, primarily dyspraxia and dyscalculia, indicating significant cognitive vulnerability within this demographic. Environmental factors, including but not limited to visual impairments, psychological distress, and dietary irregularities, such as the omission of breakfast, further exacerbated these challenges.

The results of the d2R test demonstrated significant variability. While processing speed (CCT) was largely categorized as “very high,” concentration capacity (CC) and accuracy (E%) were more heterogeneous. This finding indicates that high processing rates do not necessarily equate to better attentional quality or precision.

To support cognitive development in undernourished populations, our findings highlight the urgent need to implement school-based nutrition interventions that promote balanced meals, especially breakfast, and a stable psychosocial environment to support cognitive development. It is imperative to acknowledge the importance of addressing modifiable risk factors, particularly dietary habits and stress management, to enhance learning capacity and mental health among schoolchildren.

Overall, the study makes a significant contribution to the extant literature by offering novel insights into the relationship between dietary habits and cognitive performance among primary schoolchildren in a rural Moroccan context. This population has been underrepresented in previous research. The study combines nutritional assessments with measures of cognitive function, highlighting specific dietary patterns linked to attentional and learning outcomes. These findings not only enhance our understanding of the influence of nutrition on child development in low- and middle-income contexts, but also emphasize the importance of culturally and regionally adapted interventions to enhance both health and educational outcomes.

### 4.1 Limitations of the study

This study is limited by its cross-sectional design, reliance on self-reported data, and the absence of validated tools for assessing psychological stress. Additionally, the sample was restricted to a single rural area, which may affect the generalizability of the findings. Future studies should adopt longitudinal designs, use validated assessment tools, and include more diverse and representative samples to strengthen causal inference and generalizability.

### 4.2 Key points and relevance

The present study highlights the strong relationships among nutritional status, dietary habits, and cognitive performance among primary school students in the Khemisset region of Morocco. While undernutrition can have a detrimental effect on cognitive ability, this research provides novel regional data using BMI-for-age Z-scores and attention assessments (d2R test). These findings indicate a high prevalence of undernutrition and inadequate dietary patterns that correlate with reduced attention capacity. These results are particularly relevant to educational practice, as they suggest that the integration of school-based nutrition programs could improve learning outcomes.

### 4.3 Policy implications

This study backs the creation of public health initiatives such as nutrition education in Moroccan primary schools or free breakfast programs. Furthermore, it establishes the foundation for subsequent scientific investigations to investigate causal relationships and long-term impacts.

## Data Availability

The original contributions presented in the study are included in the article/Supplementary material, further inquiries can be directed to the corresponding author.
