# Supplementary material for: Dietary habits and cognitive performance in primary school students: a cross-sectional study in Khemisset region in Morocco
Source: Front Nutr. 2025 Oct 10;12:1643854. doi: 10.3389/fnut.2025.1643854 (PMC12549313; doi:10.3389/fnut.2025.1643854)
Supplement: Supplementary file 1 [file Data_Sheet_1.PDF]

Dietary habits and cognitive performance in primary school students: A cross-sectional study in Khemissat region in Morocco

This questionnaire is part of a doctoral research project.

Its purpose is to gather information about students' lifestyles, health, eating habits and sporting activities.

Responses are strictly anonymous and will be used for scientific purposes only.

We thank you for your sincere and valuable participation.

* Indicates required question

1. Gender *

Mark only one oval.

- Boy
- Girl

2. Age *

Mark only one oval.

- 9-10
- 11-12
- 13-14
- 15-16
- 17

3. Weight (Kg) *

____

4. Height (cm) *

____

5. Do you suffer from any health problems? *

Mark only one oval.

- Yes
- No

6. Do you have trouble vision ? *

Mark only one oval.

- Yes
- No

7. Do you practice sport ? *

Mark only one oval.

- Yes
- No

8. Do you suffer from psychological stress? *

Mark only one oval.

- Yes
- No

9. Do you have any learning difficulties? *

Mark only one oval.

- Yes
- No

10. If yes, which one

Mark only one oval.

- Diskinesia
- Reading difficulties
- Dysgraphia
- Dyscalculia

11. How many meals do you have a day? *

Mark only one oval.

- 2
- 3
- 4
- 5

12. Do you have breakfast? *

Mark only one oval.

- Yes
- No

13. When do you have your snacks? *

Mark only one oval.

- Between breakfast and lunch
- Between lunch and dinner

14. What are your daily consumed food groups? *

Check all that apply.

- Fruits and vegetables
- Dairy products
- Protein-rich products (meats, fish, eggs, etc)
- Grains et starchy foods
- Fats and oils
- Sugary and processed foods
